# A BIM-Guided Virtual-to-Real Framework for Component-Level Semantic Segmentation of Construction Site Point Clouds

**DOI:** 10.3390/s26010308

**Published:** 2026-01-03

**Authors:** Yiquan Zou, Tianxiang Liang, Jafri Syed Riaz un Nabi, Zhendong Xu, Liang Zhou, Biao Xiong

**Affiliations:** 1 School of Civil Engineering, Architecture and the Environment, Hubei University of Technology, 28 Nanli Rd, Wuhan 430068, China; zouyq@mail.hbut.edu.cn; 2 Department of Electronic Engineering, NED University of Engineering and Technology, Scheme-33, Karachi 75270, Pakistan; 3 China Construction Third Engineering Bureau Technology Innovation Industry Development Co., Ltd., 28 Sixin Avenue, Wuhan 430068, China; 4 Department of Realistic 3D Modeling, Heilongjiang Institute of Geomatics Engineering, No. 32 Cehui Road, Harbin 150086, China; 5 School of Computer Science and Artificial Intelligence, Wuhan University of Technology, 122 Luoshi Rd, Wuhan 430070, China; b.xiong@whut.com

**Keywords:** BIM-guided synthetic point clouds, virtual-to-real (V2R) semantic segmentation, synthetic point cloud generation, construction scene understanding, intelligent construction monitoring

## Abstract

LiDAR point cloud semantic segmentation is pivotal for scan-to-BIM workflows; however, contemporary deep learning approaches remain constrained by their reliance on extensive annotated datasets, which are challenging to acquire in actual construction environments due to prohibitive labeling costs, structural occlusion, and sensor noise. This study proposes a BIM-guided Virtual-to-Real (V2R) framework that requires no real annotations. The method is trained entirely on a large synthetic point cloud (SPC) dataset consisting of 132 scans and approximately $8.75\times 10^{9}$ points, generated directly from BIM models with component-level labels. A multi-feature fusion network combines the global contextual modeling of PCT with the local geometric encoding of PointNet++, producing robust representations across scales. A learnable point cloud augmentation module and multi-level domain adaptation strategies are incorporated to mitigate differences in noise, density, occlusion, and structural variation between synthetic and real scans. Experiments on real construction floors from high-rise residential buildings, together with the BIM-Net benchmark, show that the proposed method achieves 70.89% overall accuracy, 53.14% mean IoU, 69.67% mean accuracy, 54.75% FWIoU, and 59.66% Cohen’s κ, consistently outperforming baseline models. The Fusion model achieves 73 of 80 best scene–metric results and 31 of 70 best component-level scores, demonstrating stable performance across the evaluated scenes and floors. These results confirm the effectiveness of BIM-generated SPC and indicate the potential of the V2R framework for BIM–reality updates and automated site monitoring within similar building contexts.

## 1. Introduction

The accelerated digital transformation of the construction industry has positioned Building Information Modeling (BIM) as a central infrastructure element for the full life-cycle management of buildings [1]. During the construction phase, BIM is expected to serve as a digital twin of the site, enabling real-time progress monitoring, quality inspection, and safety analysis [1]. However, the inherent dynamism of construction environments and the limitations of manual measurement often impede the synchronization of BIM models with as-built site conditions, resulting in a persistent “virtual–real discrepancy” [2,3].

Three-dimensional (3D) point cloud scanning offers rich geometric and spatial information [4,5], but construction-site point clouds often suffer from severe occlusion, clutter, varying point density, and noise [6]. These limitations lead to blurred boundaries, missing data, and structural ambiguity, making reliable semantic segmentation particularly challenging [7].

Deep learning has advanced point cloud semantic segmentation with architectures such as PointNet [8], PointNet++ [9], DGCNN [10], and PCT [11]. However, their performance in real construction scenes is constrained by the scarcity of large-scale annotated 3D datasets [12]. Public datasets such as BIM-Net [13] contain only a few dozen files and about 90 million points, far from sufficient for training generalized models.

To overcome this limitation, we propose a BIM-guided Virtual-to-Real (V2R) framework for component-level point cloud semantic segmentation, relying exclusively on BIM-generated supervision. Leveraging the HELIOS++ [14] simulator, we curated an extensive synthetic point cloud (SPC) dataset comprising 132 virtual scans and approximately $8.75\times 10^{9}$ points, surpassing the scale of existing BIM-derived datasets by nearly two orders of magnitude. The dataset (~200 GB) includes component-level labels and spans diverse scanning settings, viewpoints, and densities.

Domain gaps between synthetic and real point clouds—arising from noise characteristics, point sparsity, occlusion, and surface reflectance—are addressed through both data-level and feature-level adaptation. A learnable PointAugment module [15] injects realistic distortions and occlusions into virtual scans, while channel normalization and weighted residual fusion enhance cross-domain feature alignment.

The segmentation model adopts a dual-branch fusion architecture: PointNet++ captures local geometric patterns, and PCT extracts global semantic relationships. This integration enables accurate boundary preservation and enhances robustness in geometrically complex areas.

This study aims to achieve *zero-real-annotation* component-level segmentation of real construction site point clouds *without any target-scene fine-tuning*. Experiments on a representative high-rise residential floor and the multi-scene BIM-Net benchmark demonstrate that the proposed framework achieves strong performance—including 70.89% overall accuracy, 53.14% mean IoU, 69.67% mean accuracy, 54.75% FWIoU, and 59.66% Cohen’s κ—along with high per-component IoU. These results, spanning both self-acquired site scans and a public benchmark, indicate stable BIM-to-reality transfer across different scanning conditions and scene layouts within similar residential contexts. Confusion matrix analyses show clear differentiation among walls, beams, floors, and slabs, with residual confusion mainly in the “other” class due to missing auxiliary components in virtual scenes.

Different from prior BIM-derived segmentation studies that still rely on real annotations or fine-tuning on target scans, this work formulates an *annotation-free* BIM-to-reality transfer pipeline and demonstrates that a model trained *only* on procedurally generated BIM-based SPC can achieve stable component-level segmentation on multiple real construction floors. The key enabler is a *coupled design* of scalable BIM-driven synthetic supervision (SPC) and dual-level domain bridging (learnable distortion + feature alignment) integrated into a global–local fusion network, which collectively reduces the synthetic–real discrepancy without any real labels.

The main contributions of this work are as follows:

(i) Annotation-free BIM-to-real formulation. We explicitly formulate a *zero-real-annotation* component-level segmentation setting for construction point clouds and provide an end-to-end BIM→SPC→model→real inference pipeline, avoiding any real-scene fine-tuning or pseudo-label bootstrapping.
(ii) Scalable BIM-driven synthetic supervision. We build a large-scale, reproducible BIM-derived SPC training corpus (132 scans, $∼8.75\times 10^{9}$ points) via HELIOS++ [14] with multi-parameter virtual scanning, enabling controllable density/viewpoint variations and component-level labels at negligible manual cost.
(iii) Dual-level domain bridging via global–local fusion. We propose a global–local Fusion network integrating PointNet++ and PCT, featuring learnable point cloud distortion (PointAugment) and feature-level alignment (channel normalization and weighted residual fusion). Its performance is validated on real-world scenes and the BIM-Net benchmark, where it consistently outperforms competitive baselines.

## 2. Related Work

### 2.1. Relationship Between BIM and Point Clouds

BIM technology has been widely applied in the engineering field, where the detailed modeling of existing buildings relies on accurate 3D data support [16]. Remote sensing techniques such as laser scanning (LiDAR) can capture high-precision 3D point clouds, providing rich geometric information for BIM [17]. Therefore, point cloud data has become a critical element in the BIM workflow, capturing the 3D geometry of the real environment and augmenting the model with semantic information [18]. Xiong et al. [19] underscored the pivotal role of high-fidelity point clouds in geometric inference and semantic refinement by integrating laser scans with knowledge-based rules to automate the reconstruction of indoor as-built BIM. This further emphasizes the necessity of deep integration between BIM and point clouds. A typical workflow is the “Scan-to-BIM” method, where raw point clouds collected from the physical site are converted into a digital BIM 3D model [20]. Scan-to-BIM plays an important role when original design data is missing or outdated, allowing the reconstruction of high-precision as-built models from laser-scanned point clouds [21]. However, combining massive unstructured point cloud data with the semantic models of BIM still presents many technical challenges [17], making it one of the hottest research topics in the field of digital construction.

In contrast to 2D images, publicly available 3D point cloud annotated datasets are relatively scarce. The success of deep learning depends heavily on large annotated datasets, but obtaining large-scale annotated point clouds is both labor-intensive and time-consuming [22]. Annotating 3D point clouds requires precise identification of objects in 3D space and assigning semantic labels, making the workload far greater than for 2D images, which severely limits the availability of annotated data [23]. Currently, commonly used point cloud semantic segmentation datasets are not large in scale. For example, the indoor scene dataset ScanNet v2 contains only about 1513 scan instances and covers 20 semantic categories (mainly indoor furniture objects) [24]. Specialized datasets for architectural environments, such as those for historical building heritage or mechanical and electrical pipelines, are even more scarce. This lack of datasets significantly hampers the training and evaluation of deep learning models for point clouds.

To alleviate the data shortage, researchers have started using virtual synthetic datasets to augment the training sets. For instance, Ma et al. [25] generated SPCs using BIM models to enhance indoor scene segmentation training and diversify the training data. By leveraging the complete geometric and semantic information provided by BIM, point clouds with labels can be automatically generated, thus obtaining large-scale annotated samples at low cost [22]. However, due to the domain discrepancy between SPCs and real scan point clouds, models trained with synthetic data often perform poorly on real data [26]. This “domain gap” caused by dataset differences indicates that simply increasing the amount of virtual data does not fully solve the data scarcity issue, and the models still need to be adapted for real point clouds.

These studies reveal the strong dependency of BIM workflows on high-quality point clouds and highlight the shortage of large annotated 3D datasets. However, existing works struggle with the heavy cost of real point-cloud labeling and the domain gap between synthetic and real scans. Building on these insights, our work constructs a much larger BIM-based synthetic dataset and introduces domain-adaptation mechanisms, enabling reliable real-scene segmentation without requiring any real annotations.

### 2.2. Deep Learning Approaches for Point Cloud Semantic Segmentation

In recent years, deep learning-based point cloud semantic segmentation methods have become the mainstream choice [23]. Compared to earlier methods relying on geometric rules or traditional machine learning approaches, the introduction of deep neural networks has significantly improved the accuracy of point cloud segmentation [27]. Deep learning models are capable of directly learning complex features from point clouds, enabling semantic labeling for each point. Most current point cloud segmentation studies adopt a supervised learning framework, requiring a large amount of annotated data as the training set [26]. With the advancement of technologies such as Convolutional Neural Networks (CNNs) and Transformers, point cloud segmentation algorithms have shown significant performance improvements on public benchmark datasets [28].

To address the irregularity of point cloud data, various network architectures have been proposed, including Point-Based methods that directly process raw points [3], Voxel-Based methods that voxelize point clouds [29], and Multi-View Projection-based methods [30]. Additionally, the recently introduced self-attention mechanism (Transformers) has been applied to point cloud segmentation, further enhancing feature representation capabilities and model performance [31]. For example, RandLA-Net proposed an efficient random sampling and local feature aggregation strategy, which enables point-wise semantic inference directly on large-scale point clouds [32]. Similarly, the Fast Point Transformer model introduced a lightweight self-attention layer, which improves inference speed by two orders of magnitude while maintaining accuracy. It has been reported that its inference speed on large indoor datasets is approximately 129 times faster than the previous Point Transformer model [33]. These advanced deep learning methods have significantly improved the efficiency and accuracy of point cloud semantic segmentation, representing the latest advancements in the field.

Existing deep learning methods have greatly advanced point cloud semantic segmentation through point-based, voxel-based, multi-view, and Transformer-based architectures. However, these methods still rely heavily on large annotated datasets and often generalize poorly in complex construction environments. Building on these limitations, our work removes the dependency on real annotations and introduces a BIM-guided synthetic-to-real framework with domain adaptation, enabling stable segmentation performance even under challenging real-world site conditions.

### 2.3. Point Cloud Data Annotation Challenges

High-quality, large-scale annotated datasets are difficult to obtain due to the complexity and high cost of point cloud annotation tasks. Compared to labeling 2D images, manual annotation of 3D point clouds is both time-consuming and labor-intensive, often requiring specialized domain knowledge [26]. On one hand, large point clouds typically contain millions of points, making the task of annotating each point a monumental effort [34]. On the other hand, different components in building structures often have highly similar features in point clouds, making it difficult for non-experts to distinguish between them. This not only leads to significant human resource expenditure but also increases the likelihood of errors or inconsistencies in annotations [35]. This intensive annotation workload restricts the availability of high-quality training data, thereby capping the performance potential of supervised deep learning models [36]. Additionally, Xiong et al. [37] demonstrated the potential of point clouds for target identification under noise and occlusion conditions using LiDAR in complex forest-edge environments, reflecting the challenges of feature distribution discrepancies in cross-scene point clouds.

To address annotation difficulties, the academic community has begun exploring weakly supervised and semi-supervised point cloud segmentation methods [38]. Weakly supervised methods require only partial annotations or a small number of labeled points, inferring the semantics of the remaining unlabeled points through algorithms, thus significantly reducing the amount of manual annotation [39]. For example, some studies have achieved good segmentation results on large-scale point clouds using as little as 0.1% of manually labeled data combined with pseudo-labeling strategies [23]. However, the segmentation accuracy of current weakly supervised methods still lags behind that of fully supervised training, and relying solely on a small number of annotations often does not achieve optimal results [40]. Therefore, improving the accuracy of weakly supervised methods while reducing annotation workload remains an open research challenge.

Beyond weak supervision, recent studies have investigated alternative paradigms to further alleviate or avoid manual annotation. Self-supervised representation learning has been explored to pretrain point cloud encoders using geometric consistency [41], contrastive objectives [42], or reconstruction-based tasks [43], providing transferable features for downstream segmentation with limited labeled data. In parallel, synthetic data generation have gained increasing attention, where large-scale labeled point clouds are automatically generated from virtual environments or CAD/BIM models [44]. These approaches enable precise and noise-free annotations while offering controllable variations in viewpoint, density, and occlusion patterns. However, the domain gap between synthetic and real-world point clouds—arising from sensor noise [45], incomplete geometry, and complex scene clutter—remains a major challenge, often leading to performance degradation when models are directly transferred across domains. As a result, recent research has focused on domain adaptation [46] and domain generalization techniques [47] to mitigate distribution discrepancies between synthetic and real point clouds, highlighting an active and evolving research frontier in point cloud semantic segmentation.

## 3. Methodology

At a high level, the proposed method follows a simple virtual-to-real workflow. First, a Building Information Model (BIM) is constructed to provide accurate geometric and semantic descriptions of building components. Second, the BIM model is used to generate large-scale synthetic point clouds through virtual LiDAR scanning with HELIOS++ [14], producing fully labeled training data without manual annotation. Third, the synthetic point clouds are partitioned into local blocks using a sliding-window strategy and used to train a Fusion segmentation network that combines local geometric features and global contextual information. Finally, the trained model is directly applied to real-world LiDAR scans for component-level semantic segmentation. This enables the semantic knowledge learned from virtual BIM-derived data to be effectively transferred to real construction scenes.

### 3.1. Overall Framework

**Figure 1:**
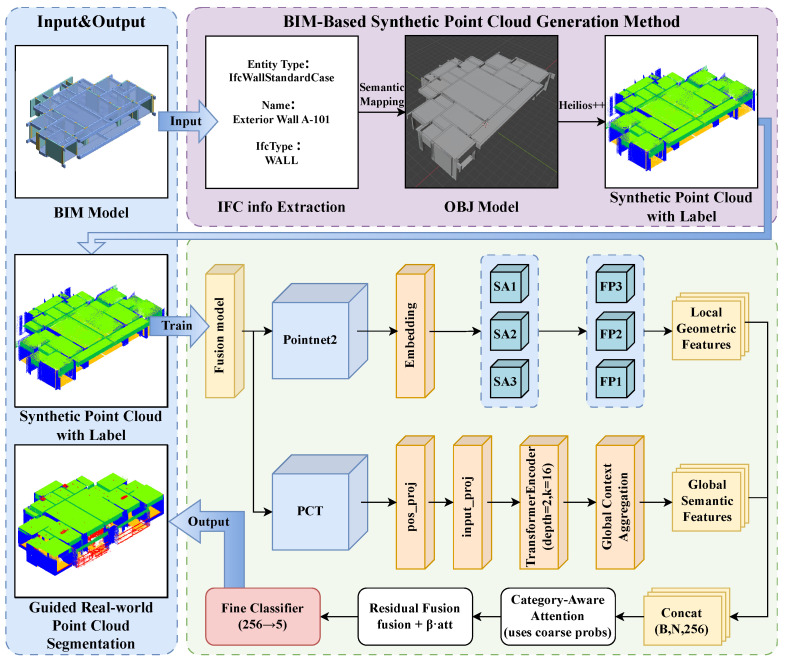
Overall workflow of the BIM-guided Virtual-to-Real point cloud semantic segmentation framework.

This study develops a complete workflow covering data generation, model training, and result evaluation to support the task of semantic understanding from BIM to real point clouds. As shown in Figure 1, the framework consists of three main stages: BIM model conversion and synthetic point cloud construction, deep model training, and semantic prediction on real point clouds.

In the data preparation stage, a Building Information Model is first created in Autodesk Revit, where components such as walls/columns, beams, slabs, and ceilings are assigned unified class codes and exported as OBJ files. HELIOS++ [14] is then used to perform multi-parameter virtual LiDAR scanning under different acquisition settings to generate semantic point clouds of varying densities. Each scanned point is automatically associated with a component-level semantic label according to its corresponding BIM instance. Subsequently, a unified preprocessing pipeline is applied, including format conversion, invalid point removal, and sliding-window partitioning, producing .npy data directly usable for training.

In the model training stage, the SPCs for constructing a segmentation network that fuses local geometric features with global semantic information. This stage focuses on network design, augmentation strategies, and optimization for training stability. Details of the model branches, fusion mechanisms, and loss functions are presented in subsequent sections and are not elaborated here.

After training, the model is applied to real LiDAR scans for component-level semantic prediction. The evaluation examines overall accuracy (OA), mean Intersection-over-Union (mIoU), frequency-weighted IoU (FWIoU), Cohen’s Kappa, and other quantitative metrics, and uses visualizations to assess model performance across different regions and component types.

Overall, the workflow integrates BIM geometry and semantic information with virtual scanning technology and deep segmentation models, forming a complete pipeline tailored for real construction environments with strong reproducibility and engineering applicability.

### 3.2. Data Preparation Stage

**Figure 2:**
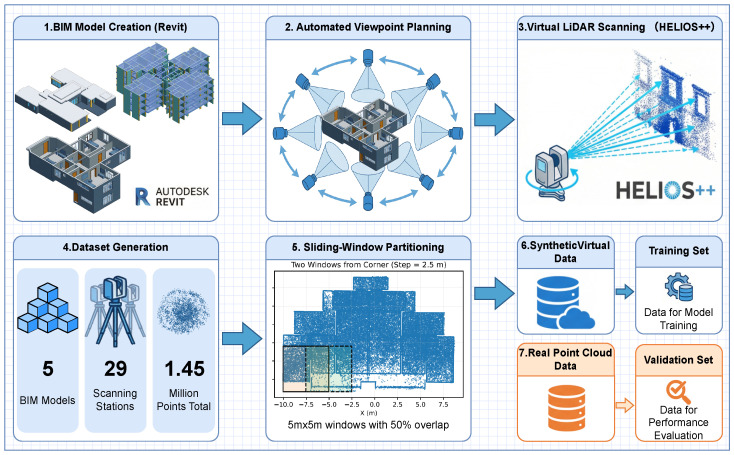
Sequential data preparation pipeline for BIM-derived synthetic point cloud generation and training data construction. The process includes: (1) BIM model creation in Autodesk Revit; (2) automated viewpoint planning for scanner placement; (3) virtual LiDAR scanning using HELIOS++ [14]; (4) synthetic dataset generation under multiple scanning configurations; (5) sliding-window partitioning with fixed window size and overlap; and (6) final organization of synthetic and real point cloud data for training and validation.

An overview of the entire data preparation pipeline is illustrated in Figure 2. The workflow employs a sequential, modular architecture that encompasses BIM creation, automated viewpoint planning, and virtual LiDAR scanning, followed by synthetic data generation and sliding-window partitioning to prepare datasets for subsequent training and evaluation. This figure provides a global reference for the individual steps detailed in the following subsections.

This stage is designed to bridge BIM models and real construction point clouds in a controlled manner by using BIM as a source of complete geometric and semantic priors instead of relying on limited real annotations. Virtual LiDAR scanning enables systematic control of point density, viewpoints, and coverage, while automated viewpoint planning ensures consistent visibility across scenes. Sliding-window partitioning with overlap is adopted to stabilize local geometric learning and reduce the impact of density imbalance in large-scale scenes. Together, these choices yield a scalable and domain-relevant synthetic training corpus, allowing the segmentation model to focus on structural semantics and supporting annotation-free virtual-to-real transfer.

This study refers to the typical building model in the public BIM-Net dataset [13] and uses a high-rise residential building in Guangzhou as the prototype. A complete BIM was created in Autodesk Revit, where the primary components (walls/columns, beams, slabs, ceilings, etc.) were assigned unified class codes and semantic labels. The geometric structure and component configuration were based on actual construction blueprints to ensure the virtual scene accurately represents the complexity and spatial characteristics of the real building.

Following the BIM-Net convention, different floors or structural zones extracted from BIM models are assigned unique scene identifiers (e.g., 1px, 7y3, ac2, vvo), each corresponding to a distinct building–floor combination. As summarized in Table 1, the proposed framework is trained exclusively on 132 synthetic SPC scenes generated from 22 BIM models under six virtual scanning configurations, while a subset of 16 real-world BIM-Net scenes is reserved solely for validation and testing. All real scenes originate from different buildings and floors, with no structural overlap between scenes, ensuring a clear separation between training and evaluation data and supporting reproducible cross-scene performance assessment.

**Figure 3:**
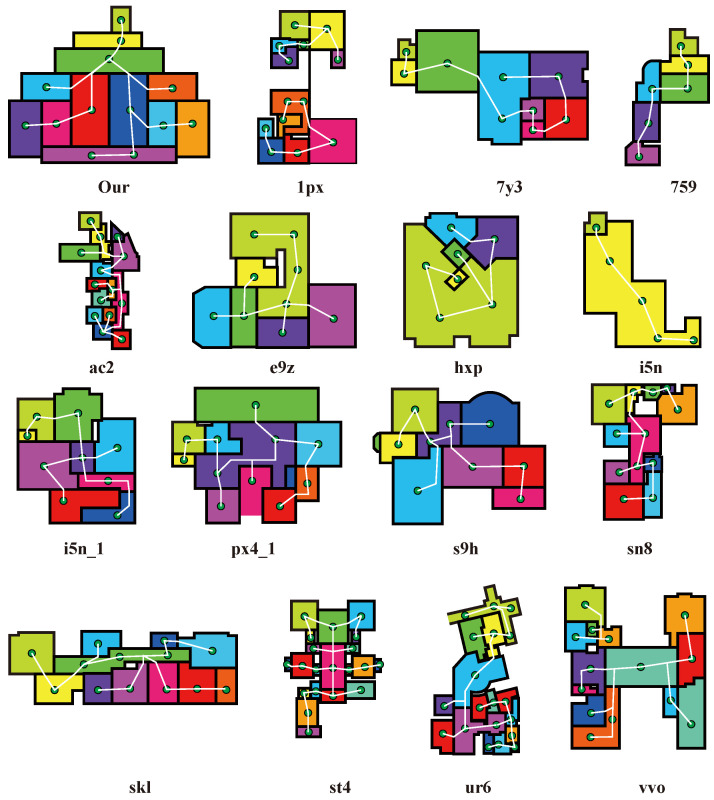
Synthetic LiDAR Scanning Network from BIM Model. The green points represent the locations of the virtual scanners. Scene IDs shown in the figure (e.g., 1px, 7y3, 759, ac2, vvo) follow the original BIM-Net naming convention and denote different building floors or structural zones with varying geometric complexity and occlusion characteristics.

In addition, the virtual LiDAR scanner stations were not manually specified. Instead, their locations (visualized as green points in Figure 3) were generated using an automated viewpoint planning strategy inspired by the VF-Plan framework proposed by Xiong et al. [48]. This method evaluates geometric visibility, occlusion, and coverage requirements to produce an optimized set of scanner poses. The automatically planned stations ensure that the synthetic LiDAR captures the building components with coverage and completeness comparable to practical terrestrial laser scanning.

In the virtual scanning phase, ground-based LiDAR simulation was implemented using the HELIOS++ platform. The scanners were evenly arranged according to the building layout to capture comprehensive and complementary point cloud data. To increase the diversity of the SPC and simulate different equipment and acquisition conditions, six typical parameter combinations were designed based on Zou et al.’s study [49] on the balance between scanning efficiency and point cloud density in construction scenarios (Table 2). By adjusting the pulse frequency and scanning frequency, the point cloud density and resolution were controlled. Other parameters (scan angle $90^{{}^{\circ}}$, pitch angle range $-90^{{}^{\circ}}∼90^{{}^{\circ}}$, rotation speed $15^{{}^{\circ}}$/s) were kept constant to ensure the experiment’s controllability and reproducibility. Following the “optimal density range” principle outlined in the literature, we selected a point density range close to the best density for inclusion in the training set, ensuring the expression of geometric details while avoiding redundant sampling, thus improving the model’s generalization across different density scenarios.

The raw point clouds output by HELIOS++ are stored in .XYZ format, containing spatial coordinates, reflectance intensity, and component IDs. Semantic labels are automatically generated based on BIM category information, mapping components such as walls/columns, beams, ceilings, floors, and others to labels 1–5, enabling point-level semantic annotation without manual intervention.

Compared with the BIM-Net dataset, which contains just over twenty files and 90.4 million points in total, our synthetic dataset is significantly larger in scale. Statistical analysis shows that 132 SPC files were generated, comprising approximately $8.75\times 10^{9}$ points in total—nearly two orders of magnitude larger than BIM-Net. Each file contains an average of $6.63\times 10^{7}$ points, and the overall data volume reaches roughly 200 GB. Such a large-scale dataset provides a far more comprehensive representation of structural geometry and ensures sufficient diversity and density to support high-capacity deep learning models and reliable cross-scene generalization studies.

**Figure 4:**
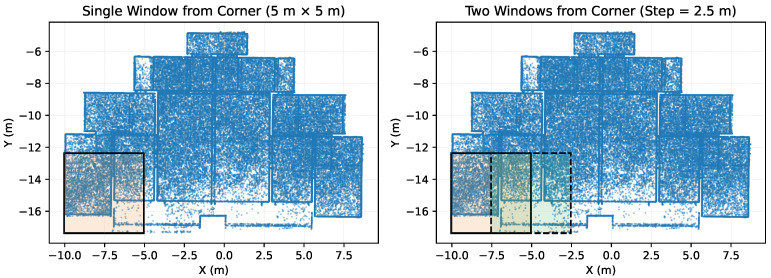
Sliding-window partitioning strategy for point cloud preprocessing. Blue points represent the input point cloud, while shaded areas mark individual window coverage and the spatial overlap introduced by the 2.5 m stride.

To help the model learn more stable geometric structures at the local scale, both virtual and real point clouds undergo spatial partitioning using a sliding window approach during a unified preprocessing stage. The partitioning process starts at the corner of the scene’s bounding box, with each window fixed at a 5m×5m 2D projection area. The window slides along the *X*-axis with a stride of 2.5m, achieving approximately 50% spatial overlap. Figure 4 shows a top-down view of this partitioning approach: the left side shows the coverage of a single window, while the right side illustrates the overlapping relationship between two adjacent windows. Blue points represent the original point cloud, the light-colored rectangles indicate the corresponding window ranges, and black or dashed borders indicate the window positions.

This overlapping local partitioning approach helps maintain geometric continuity across blocks, improving the stability of boundary region samples and mitigating class imbalance caused by point cloud point density. The sliced data is normalized into a uniform [N,4] structure (x,y,z,c), facilitating batch loading and model training. During inference, predictions are first obtained independently for each sliding window. For points that appear in multiple overlapping windows, the final semantic label is determined by aggregating the predicted class probabilities across all corresponding windows and assigning the class with the highest averaged probability. This simple probability-based fusion strategy ensures consistent predictions in overlapping regions and avoids boundary artifacts when reconstructing the full-scene segmentation.

### 3.3. Model Architecture Design

There are significant differences between SPC and real scan point clouds in terms of noise levels, point density distribution, and occlusion conditions, which directly lead to a shift in their semantic space. Existing point cloud segmentation models, when trained under fully supervised or single-domain settings, are often insufficient to handle such discrepancies, as they tend to overfit domain-specific feature distributions and exhibit limited robustness under cross-domain deployment. To address this issue, we propose a segmentation framework driven by both local geometric encoding and global semantic modeling, which is motivated by the observation that purely local or purely global representations alone cannot adequately compensate for the combined effects of noise, density variation, and structural occlusion encountered in real construction scenes. aiming to maintain stable recognition performance in real construction environments while fully relying on BIM-generated virtual data for training.

The overall architecture of the model is shown in Figure 1, consisting of two branches: PointNet++ and PCT. PointNet++ extracts local geometric features through hierarchical sampling and neighborhood aggregation mechanisms, capturing the boundary structures, contact relationships, and fine-scale forms of components. These features exhibit high consistency between virtual and real point clouds. Meanwhile, the PCT branch, centered around the self-attention mechanism, establishes long-range point dependencies through multi-head feature interaction, forming a global understanding of the building components’ overall layout and topological relationships. This global semantic modeling plays a crucial role in resolving feature breaks caused by varying scan densities, viewpoint changes, and local occlusions.

During the feature fusion phase, the network aligns the outputs of both branches into a unified semantic space and adjusts the relative contributions of local and global features through channel normalization and weighted residual connections. This mechanism adaptively mitigates distribution differences between virtual and real point clouds, preventing one branch from being overemphasized during training and reducing the accumulation of cross-domain bias. The fused features are then mapped linearly, and the classification head outputs point-level semantic labels for component recognition in real-world scenes.

Let $F\in R^{B\times N\times D}$ denote the concatenated fusion feature, with $F=[F^{\mathrm{PN}2},F^{\mathrm{TR}}]$ and D=2E. A lightweight coarse classifier produces point-wise coarse logits $Z^{c}\in R^{B\times N\times C}$, which are converted to coarse probabilities

(1) $$P^{c}=\mathrm{Softmax}\;\left(Z^{c}/\tau \right).$$

Here, *B* denotes the batch size, *N* the number of points per sliding window, *E* the feature dimension of each branch, *C* the number of semantic classes, and τ a temperature parameter.

By combining the local geometric stability of PointNet++ with the global semantic consistency of PCT, this structure establishes a transferable feature representation between virtual training data and real application scenarios. This enables the model to maintain strong cross-domain generalization ability, even when real annotations are not available.

### 3.4. Training and Optimization Strategies

The model training employs distributed multi-GPU parallelism and automatic mixed precision computation to enhance efficiency while ensuring numerical stability. The AdamW optimizer is used, combined with OneCycleLR for learning rate scheduling, and the overall training process follows the setup of mainstream deep learning frameworks. To alleviate training bias caused by class imbalance in the building point cloud, we introduce a class-weighting mechanism during the data sampling phase and use Exponential Moving Average (EMA, α=0.99) during the optimization phase to smooth parameter updates, thereby improving convergence stability and generalization ability.

For the loss function, we adopt a combination of cross-entropy and Focal Loss to balance classification stability and the ability to identify hard samples. The standard cross-entropy loss $L_{\mathrm{ce}}$ is defined as:

(2) $$L_{\mathrm{ce}}=-\frac{1}{N}\sum_{i=1}^{N}\sum_{c=1}^{C}y_{i,c}\log\left(p_{i,c}\right),$$

To enhance the model’s focus on low-confidence samples, Focal Loss is introduced:

(3) $$L_{\mathrm{focal}}=-\frac{1}{N}\sum_{i=1}^{N}{\left(1-p_{i,y_{i}}\right)}^{\gamma }\log\left(p_{i,y_{i}}\right),$$

where the focusing parameter γ=2.0 has shown stable performance across multiple experiments. The final loss is defined as:

(4) $$L=\left(1-\lambda \right)L_{\mathrm{ce}}+\lambda L_{\mathrm{focal}},\;\lambda =0.3,$$

This combination maintains good classification performance under noise interference and sample scarcity conditions.

In terms of data augmentation, in addition to conventional geometric perturbations such as random rotation, scaling, translation, and jittering, we further introduce an adaptive augmentation network based on PointAugment. This network uses a joint constraint of geometric consistency loss ($\lambda_{\mathrm{geom}}=0.1$) and consistency loss ($\lambda_{\mathrm{cons}}=0.2$) to adaptively generate samples that are more consistent with the feature distribution of real scans, significantly enhancing the domain adaptation ability between virtual and real point clouds. To increase the model’s robustness to local structures, we also introduce a probabilistic spatial occlusion mechanism (occlusion ratio of 0.2) during training to prevent the model from over-relying on prominent region features.

For completeness, the main training hyperparameters are summarized here, while additional low-level implementation details are provided in the Appendix A and Appendix B.

For hyperparameter settings, the initial learning rate is set to $2\times 10^{-3}$, weight decay is $1\times 10^{-4}$, the batch size is 8, gradient accumulation steps are 4, total training epochs are 100, the warm-up phase lasts for the first 15 epochs, the maximum number of points per sample is 8192, and coordinate normalization is enabled. With the combined effect of these optimization strategies, the model achieves stable convergence and significant cross-domain segmentation performance improvement across multiple scenarios.

### 3.5. Model Validation and Performance Analysis

#### 3.5.1. Visualization and Qualitative Analysis

During the inference phase, the preprocessed test samples (.npy) are input into the trained Fusion model for component-level semantic prediction. The network performs forward propagation on the feature vector of each point, outputs class probabilities, and applies Softmax normalization. The final label is obtained by taking the argmax of the probabilities, resulting in a segmentation output that aligns with the spatial arrangement of the input point cloud. All inference processes are conducted under fixed hardware and random seed conditions, without introducing additional data augmentation or post-processing, ensuring objective and reproducible evaluation.

To visually present the recognition results, a unified color mapping is applied to five target categories: walls/columns (blue), beams (dark green), ceilings (green), floors (orange), and others (red). The predicted results are then projected back into 3D space and compared with the ground truth labels. Additionally, key regions, such as beam-column connections, wall-panel junctions, floor boundaries, and wall corners, are selected for local zoomed-in visualizations to examine boundary continuity, semantic consistency, and detail fidelity under complex geometries and occlusion conditions.

#### 3.5.2. Quantitative Evaluation Metrics

To comprehensively evaluate the overall performance of various models in multi-class semantic recognition tasks, five commonly used metrics are selected for comparison: OA, mIoU, mean Accuracy (mAcc), FWIoU, and Cohen’s Kappa coefficient (κ). Let $N_{c}$ denote the number of categories, and ${\mathrm{TP}}_{i}$, ${\mathrm{FP}}_{i}$, and ${\mathrm{FN}}_{i}$ represent the true positives, false positives, and false negatives of class *i*, respectively. The number of samples in class *i* is $n_{i}$, and the total number of samples is $N=\sum_{i=1}^{N_{c}}n_{i}$. The metrics are defined as follows.

Overall Accuracy (OA) is given by:

(5) $$\mathrm{OA}=\frac{\sum_{i=1}^{N_{c}}{\mathrm{TP}}_{i}}{N}.$$

The Intersection-over-Union (IoU) for class *i* and the mean IoU (mIoU) are defined as:

(6) $${\mathrm{IoU}}_{i}=\frac{{\mathrm{TP}}_{i}}{{\mathrm{TP}}_{i}+{\mathrm{FP}}_{i}+{\mathrm{FN}}_{i}},$$

(7) $$\mathrm{mIoU}=\frac{1}{N_{c}}\sum_{i=1}^{N_{c}}{\mathrm{IoU}}_{i}.$$

Mean Accuracy (mAcc) measures the average recall across classes and is unaffected by category frequency imbalance. It is defined as:

(8) $$\mathrm{mAcc}=\frac{1}{N_{c}}\sum_{i=1}^{N_{c}}\frac{{\mathrm{TP}}_{i}}{{\mathrm{TP}}_{i}+{\mathrm{FN}}_{i}},$$

where $\frac{{\mathrm{TP}}_{i}}{{\mathrm{TP}}_{i}+{\mathrm{FN}}_{i}}$ denotes the recall of class *i*. mAcc reflects the model’s ability to recognize fine-grained categories and is particularly suitable for building point clouds where category imbalance and long-tail classes are common.

Considering class imbalance, the Frequency Weighted IoU (FWIoU) is defined as:

(9) $$\mathrm{FWIoU}=\sum_{i=1}^{N_{c}}\frac{n_{i}}{N}\;\cdot \;{\mathrm{IoU}}_{i}.$$

To quantify the agreement between predictions and ground truth, Cohen’s Kappa coefficient is further adopted:

(10) $$\kappa =\frac{p_{o}-p_{e}}{1-p_{e}},$$

where $p_{o}$ denotes the observed agreement (i.e., OA) and $p_{e}$ denotes the expected random agreement derived from the marginal distributions of predicted and true labels.

For consistent visual comparison across metrics, all evaluation indicators are normalized to the range [0,1] using max normalization:

(11) $$v^{\prime }=\frac{v}{\max(v)},$$

where *v* is the original metric value and $v^{\prime }$ is the normalized result. This normalization is solely for visualization and does not affect the original metric calculations or evaluation conclusions.

Based on the above evaluation framework, we conduct systematic comparisons of multiple network models across multi-parameter scanning settings and multi-floor structural scenarios, enabling a comprehensive analysis of their overall recognition performance, cross-domain generalization, and robustness.

#### 3.5.3. Component-Specific Evaluation Metrics

Building on the overall performance metrics, this study further analyzes the model’s recognition ability at the component level to reveal the performance differences across categories. For the five components—walls/columns (Wall/column), beams (Beam), ceilings (Ceiling), floors (Floor), and others (Other)—the IoU for each category is calculated. The definition of component-level IoU is consistent with ${\mathrm{IoU}}_{i}$ in Equation (6), specifically as follows:

To facilitate a horizontal comparison of model performance across different component categories, we apply the same max normalization method as in Equation (11) to rescale the component-level IoU:

(12) $${\mathrm{IoU}}_{i}^{\prime }=\frac{{\mathrm{IoU}}_{i}}{\max(\mathrm{IoU})}.$$

Compared to the overall mIoU, the component-level IoU more directly reflects the model’s robustness across different structural features. In real-world construction scenarios, planar components (e.g., floors, ceilings) with regular shapes and high point cloud density are typically easier to recognize, while elongated components (e.g., beams, columns) are more prone to misclassification due to occlusion, blurred edges, and scale variation. Analyzing component-level metrics not only reveals the advantages and disadvantages of different feature modeling mechanisms (such as global Transformer and local geometric convolution) across component categories but also helps verify the effectiveness of the BIM-guided fusion strategy proposed in this study for recognizing complex geometric structures.

#### 3.5.4. Classification Error and Confusion Analysis

To further analyze the recognition bias and confusion between different component categories, we construct a confusion matrix $C\in N^{N_{c}\times N_{c}}$, where $C_{ij}$ represents the number of points from the true class *i* that are predicted as class *j*. By normalizing the matrix along rows and columns, we obtain the recall and precision distributions for each class, which are used to evaluate the model’s performance in within-class recognition and inter-class differentiation.

Furthermore, to quantify the intensity of class-level confusion, we compute the correlation matrix based on the normalized confusion matrix. This matrix evaluates the systematic confusion relationships between different categories. It reflects the statistical correlation of categories in prediction error patterns and helps identify clusters of classes that are frequently confused with each other. This analysis reveals typical sources of errors when the model encounters spatially similar structures, closely related geometric features, or uneven point cloud density.

Confusion analysis provides an intuitive understanding of the level of confusion between component categories. In building point clouds, differences in morphology and spatial distribution between components are significant. Planar components, such as floors and ceilings, have regular geometry, while elongated components like beams and columns are complex in shape and have blurry boundaries, often leading to misclassification when neighborhood features are unclear. Additionally, occlusion, noise, and local feature degradation further exacerbate inter-class confusion.

In summary, by combining the analysis of the confusion matrix and the correlation matrix, we can systematically uncover the error patterns between specific categories. This provides a foundation for subsequent feature optimization, spatial relationship constraints, or hierarchical semantic prior designs. By integrating BIM topological structure information, we can further explore reducing inter-class confusion using the spatial dependencies between components, thereby improving the consistency and robustness of segmentation results.

## 4. Experimental Results and Analysis

### 4.1. Experimental Design and Ablation Rationale

Although the proposed Fusion model integrates multiple feature modeling mechanisms, its experimental evaluation inherently provides a clear module-level ablation analysis. Specifically, the Fusion architecture is constructed by combining two representative paradigms in point cloud semantic segmentation: local geometric modeling (PointNet++) and global contextual modeling (PCT).

Accordingly, reporting the performance of PointNet++, PCT, and their fused variant under an identical training and inference configuration already constitutes a core architectural ablation. This comparison directly reflects three complementary settings: local-only modeling, global-only modeling, and global–local cooperative modeling. By analyzing the performance differences among these settings, the contribution of the fusion design can be quantitatively isolated without introducing additional artificial ablation variants.

In addition, PointAugment [15] is not treated as a novel algorithmic module in this study, but as a standardized data augmentation strategy [50,51]. To ensure fair comparison, it is consistently applied to all baseline methods and model variants, rather than being selectively enabled.

The effectiveness of PointAugment has been systematically validated in prior work and widely adopted in recent point cloud segmentation studies. Therefore, it is regarded as part of the unified experimental setup rather than an independent variable requiring separate ablation. This design choice allows the experimental analysis to focus on the architectural contribution of the proposed Fusion framework itself.

### 4.2. Component Recognition Results

**Figure 5:**
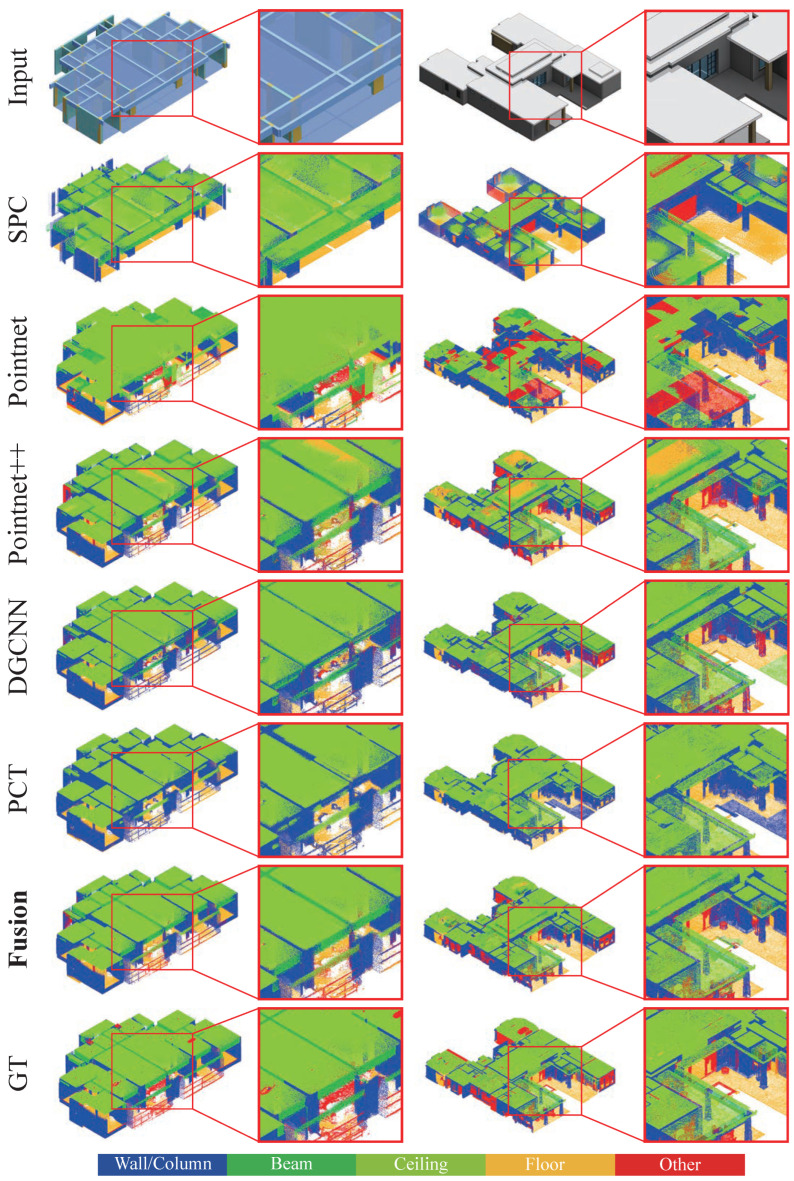
Comparison of semantic segmentation results from multiple models on real construction point clouds. The vertical columns show input, SPC, PointNet, PointNet++, DGCNN, PCT, Fusion model, and GT. The horizontal rows display two samples and their zoomed-in regions. The Fusion model demonstrates superior performance in terms of local geometric details, component boundary continuity, and semantic consistency.

To visually assess the semantic segmentation performance of each model in real construction scenarios, we selected typical floor point cloud segments and compared the prediction results of PointNet [8], PointNet++ [9], DGCNN [10], PCT [11], and the proposed Fusion model, against manually labeled ground truth (GT). Figure 5 shows the input point cloud, the outputs of each model, and zoomed-in regions for visualization. The color map corresponds to five component categories: Wall/column, Beam, Ceiling, Floor, and Other.

From overall appearance to local details, the performance differences across models in component boundaries, complex geometric regions, and occlusion areas are evident. Traditional models like PointNet and DGCNN still exhibit large misclassifications or noisy spots even in relatively simple ceiling and floor regions, indicating limited stability in scenarios with variations in point density and simpler spatial structures. PCT improves semantic consistency in large-scale continuous regions, but the distinction between “wall/column-other” categories remains unclear, with some wall-adjacent objects or noise points erroneously classified into the wall region, leading to fuzzy local boundaries.

The relatively best-performing PointNet++ demonstrates stable recognition ability for major structural components but still exhibits some misclassification and under-segmentation in complex ceiling-floor junctions and the Other category, particularly in areas with large viewpoint changes or sparse point clouds.

In contrast, the Fusion model exhibits more stable boundary continuity and semantic consistency in most scenes. In regions such as beam-column connections, wall-panel intersections, and complex ceiling structures, Fusion’s predictions are often closer to the ground truth, with a noticeable reduction in noise points. In real construction sites, where non-structural components (e.g., furniture, curtains, temporary material piles) are abundant, Fusion model also shows better recall for the Other category and handles common cross-domain issues in real point clouds, such as uneven density, occlusion, and reflection changes. This indicates that the collaborative mechanism between the global Transformer and local PointNet++ branches has strong domain adaptation capabilities in complex components and dynamic environments.

The above results indicate that the architecture combining global and local features helps alleviate the inter-class confusion issues caused by BIM virtual data training, resulting in more stable component recognition in real-world scenarios.

### 4.3. Overall Performance Metrics Comparison

On the complete dataset, the proposed Fusion model achieves an overall accuracy of 70.89%, a mean IoU of 53.14%, a mean accuracy of 69.67%, a FWIoU of 54.75%, and a Cohen’s κ of 59.66%. These results demonstrate the model’s strong recognition capability, class separability, and robustness under diverse scanning conditions.

**Figure 6:**
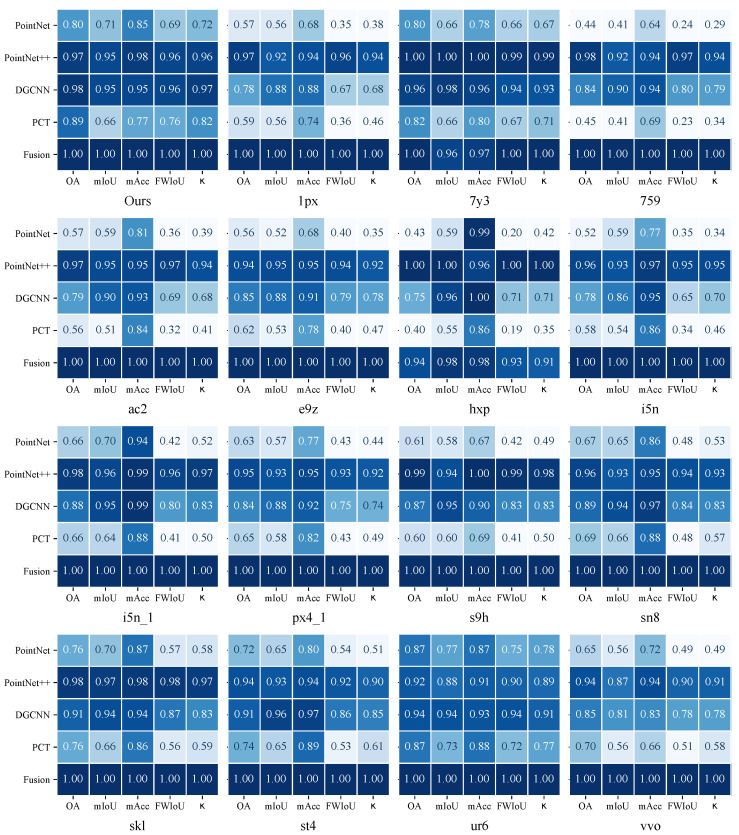
Normalized overall semantic segmentation metrics (OA, mIoU, mAcc, FWIoU, κ) across 16 scenes and five models. Darker colors indicate higher performance.

Figure 6 further provides a detailed comparison of the five normalized performance metrics (OA, mIoU, mAcc, FWIoU, and κ) across 16 scanning scenes for the five representative semantic segmentation models: PointNet, PointNet++, DGCNN, PCT, and the proposed Fusion model. All metrics are normalized following Equation (11), enabling consistent comparison across scenes and models.

A statistical examination of the normalized matrix shows that the Fusion model achieves 73 highest values out of 80 scene–metric combinations, outperforming all other models in more than 90% of the evaluation entries. This result indicates that the Fusion model maintains consistently high performance across a wide range of scenes and evaluation metrics.Such consistency suggests stable accuracy and reliable geometric discrimination under varying structural layouts, point densities, and scanning viewpoints.

Among the baseline methods, PointNet++ and DGCNN exhibit the most stable behavior, whereas PointNet and PCT show noticeable performance drops in more challenging scenes such as “1px,” “s9h,” and “skl,” where point sparsity or complex component geometry increases class ambiguity. These variations are most prominent in mIoU and mAcc, which are more sensitive to class imbalance and local structural complexity.

In contrast, the Fusion model consistently achieves the highest or near-highest normalized scores across almost all scene–metric combinations. Its integration of global contextual modeling, local geometric encoding, and category-aware attention strengthens its robustness to occlusion, sampling unevenness, and viewpoint variability.

The heatmap comparison demonstrates that the proposed Fusion model delivers superior cross-scene stability and discriminative capability. Its 73 leading entries, together with the strong performance observed on the complete dataset, highlight the effectiveness of the fusion strategy and its suitability for real construction-site semantic segmentation tasks.

Beyond normalized visualization, the original metric values reveal that the proposed Fusion model achieves consistent and non-trivial absolute improvements over the strongest baseline across multiple scenes. For example, on the *759* scene, Fusion improves mIoU from 51.1% (PointNet++) to 55.5%, corresponding to a +4.4 percentage-point gain, which is the largest absolute mIoU improvement observed among all evaluated scenes. Meanwhile, Cohen’s κ increases from 54.9 to 58.2 (+3.3 pp), indicating substantially improved agreement between predictions and ground truth. These absolute gains confirm that the dominance observed in the normalized heatmap reflects meaningful performance improvements rather than a visualization artifact.

### 4.4. Component-Level Performance Comparison

Building upon the overall performance evaluation, we further analyze the Fusion model’s recognition capability at the component level across different categories. At the component level, the Fusion model generally attains the highest or near-highest IoU values across most scene–component combinations, while maintaining consistently competitive performance in the remaining cases. This pattern indicates stable component discrimination under diverse structural layouts and scanning conditions, without relying on scene-specific tuning.

**Figure 7:**
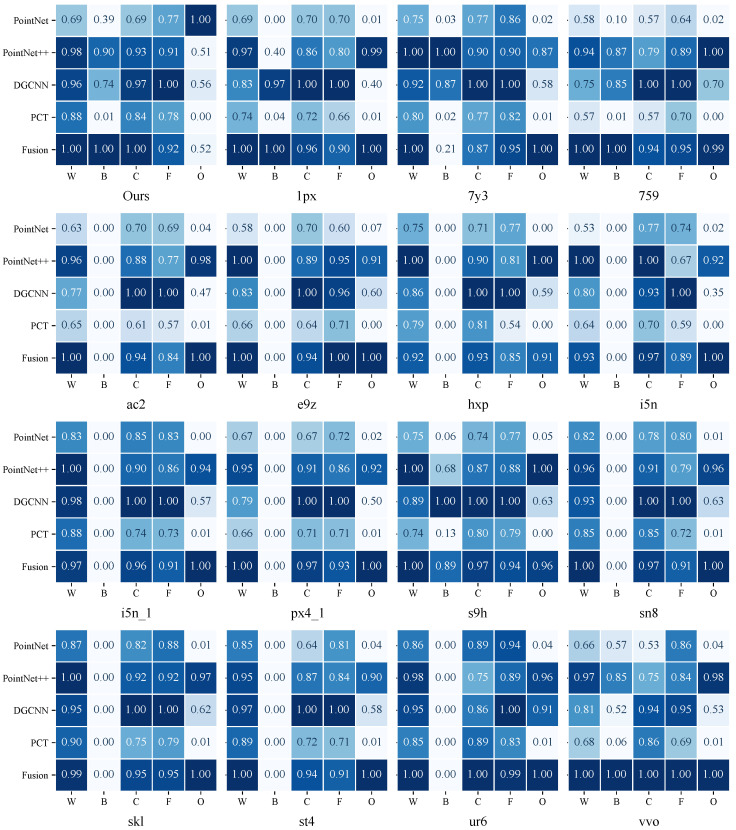
Normalized IoU comparison of five component categories (Wall/Column (W), Beam (B), Ceiling (C), Floor (F), Other (O)) across 16 scenes and five models. Darker color indicates higher performance.

Figure 7 presents the normalized IoU performance of five major components—Wall/Column, Beam, Ceiling, Floor, and Other—across 16 scenes. For consistent cross-scene comparison, all IoU values were normalized using the max-normalization method defined in Equation (11). It is important to note that in some scenes, beam components do not exist (e.g., certain floor scans do not include beam elements). As a result, all models achieve an IoU of 0 for the Beam category in those scenes, which reflects the absence of the component rather than a model performance issue.

From the cross-scene patterns, the Fusion model maintains relatively high normalized scores across most component categories, demonstrating strong consistency. For core structural components such as Wall/Column and Floor, the model typically reaches higher normalized IoU values. This advantage can be attributed to its dual-branch architecture: the global Transformer branch captures broader spatial dependencies, while the local PointNet++ branch provides fine-grained geometric cues, jointly improving recognition stability.

For categories more susceptible to noise, occlusion, or uneven sampling—such as Ceiling and Other—the Fusion model continues to exhibit robust performance. Compared with PointNet and PCT, it demonstrates better adaptability in ambiguous regions or areas with strong local geometric variation, especially near wall-panel junctions, beam–column connections, and other structurally complex interfaces, where it preserves more balanced boundary predictions.

For elongated components such as Beams, the Fusion model consistently achieves higher normalized scores in scenes where beam elements are present. The performance of PointNet and PCT fluctuates considerably in this category, whereas the Fusion model remains stable, suggesting that its joint global–local geometric representation is particularly suitable for components with scale variability and local sparsity.

The component-level heatmap collectively indicates that the Fusion model delivers strong adaptability across categories, stable cross-scene performance, and clear advantages for components with geometry-sensitive characteristics. Subsequent sections further examine misclassification patterns and structural distinctions through confusion matrix analysis and feature visualization.

### 4.5. Classification Error and Confusion Relationship Analysis

**Figure 8:**
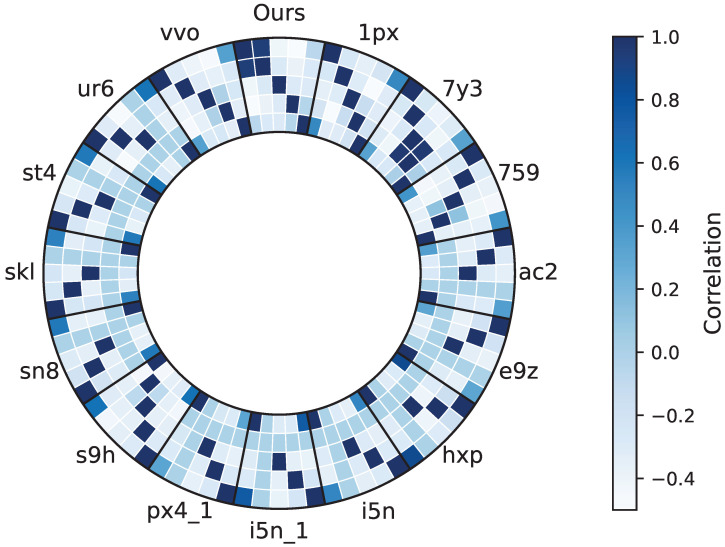
Cross-scene component-level correlation ring map. Darker color indicates higher correlation, while lower correlation reflects stronger inter-class separability.

To further understand the model’s discriminative capability across different component categories, we construct an inter-class feature correlation matrix based on the prediction results of each scene, and visualize it in a circular (ring-shaped) layout as shown in Figure 8. Higher correlation values typically indicate that categories are closer in feature space and thus more prone to confusion, whereas lower correlations imply clearer decision boundaries learned by the model.

From the overall distribution, the class-wise correlation patterns are generally consistent across models in most scenes. The Fusion model, however, tends to exhibit lower cross-category correlations among multiple component groups (e.g., Wall/Column, Beam, Ceiling, Floor), indicating that the Fusion model produces clearer inter-class separation in the feature space, thereby reducing the likelihood of mutual misclassification.

Local dark regions can still be observed in certain scenes—such as between Beam-Floor or Wall-Other—suggesting that these categories remain feature-similar under specific conditions, which can increase classification difficulty. For example, in scenes such as *1px* and *s9h*, the Beam–Floor pair exhibits noticeably higher correlation values in Figure 8. Visual inspection reveals that these errors mainly occur near beam–slab junctions, where elongated beam elements are partially occluded or sparsely sampled and appear locally planar. In practice, this leads to beams being partially absorbed into floor regions, which could result in missing or fragmented beam components in downstream scan-to-BIM reconstruction and quantity take-off workflows.

This phenomenon mainly stems from two factors. First, certain component pairs are naturally adjacent in building structures, making their local geometric features difficult to separate under partial occlusion or sparse sampling. Second, the data characteristics of virtual and real scenes differ substantially.Virtual scenes contain relatively simple component types and lack elements commonly present in real construction sites, such as curtains, temporary obstructions, or other non-structural objects. A representative case can be observed in scenes such as *skl* and *sn8*, where the Wall/Column–Other correlation is relatively high.In these scenes, non-structural objects (e.g., temporary materials, stacked equipment, or furniture) are often located close to wall surfaces and share similar vertical geometry. As a result, the model occasionally assigns these objects to the Wall/Column category. From a scan-to-BIM perspective, this leads to spurious wall extensions or false-positive wall regions, which may degrade as-built model accuracy and affect subsequent spatial analysis.

From a cross-scene perspective, the correlation patterns vary rather than being uniform, reflecting differences in scanning viewpoints, point density, and local occlusion conditions. The Fusion model maintains relatively balanced inter-class correlation distributions in most scenes, suggesting that the complementarity between its global Transformer branch and local geometric feature extraction modules enables stable category discrimination under diverse scanning conditions.

Overall, the correlation ring map highlights the Fusion model’s strengths in inter-class feature separability, cross-scene stability, and handling of geometrically adjacent component regions. At the same time, the highlighted Beam-Floor and Wall-Other confusion cases provide concrete insights into the remaining failure modes, indicating that future improvements should focus on enhancing boundary awareness at structural junctions and enriching non-structural component representations to further reduce ambiguity in real-world construction environments.

## 5. Discussion

To facilitate semantic understanding from BIM to real-world point clouds, this study develops an integrated framework encompassing virtual LiDAR scanning, sliding-window preprocessing, and Fusion model training. When trained solely on BIM-generated synthetic data, the Fusion model achieves robust transfer performance across diverse real-world scenes, consistently outperforming classical baselines in metrics such as mIoU and κ. This effectiveness stems from our dual-level strategy to mitigate domain gaps through learnable PointAugment and weighted feature alignment. Nevertheless, practical BIM-reality discrepancies—such as construction tolerances, design intent deviations, and unmodeled site elements—remain inherent challenges that can introduce semantic ambiguity at structural junctions. Quantifying the impact of these geometric inconsistencies is essential for enhancing the reliability of the proposed framework in high-fidelity automated construction monitoring.

When virtual point clouds exhibit sufficient structural and geometric consistency with real scenes, models trained solely on synthetic data can achieve competitive performance on real scans, as reported in prior studies [25,52,53]. The experimental results in this work, obtained under the BIM-derived construction site scenario, align well with these findings and further confirm that virtual scanning–generated point clouds can encode transferable component-level semantics, supporting effective virtual-to-real semantic transfer despite inevitable domain discrepancies.

Compared with conventional point cloud training strategies that rely on extensive on-site LiDAR acquisition and labor-intensive manual annotation, the proposed framework substantially reduces the dependency on repeated field scanning and real data labeling. By leveraging BIM-derived synthetic point clouds as the sole source of supervision, effective model training can be conducted prior to or independently of large-scale real data collection. From a practical perspective, the computational cost of synthetic data generation remains moderate: even under the highest configuration settings adopted in this study, generating a complete synthetic point cloud for a single BIM model requires no more than 30 min, which is significantly more efficient than repeated on-site scanning and manual data preparation. Moreover, the proposed framework does not introduce a noticeable increase in training or inference resource requirements compared with representative deep learning–based point cloud segmentation methods, as both training and block-wise inference follow standard GPU-based pipelines.

Despite the absence of real annotations, the proposed Fusion model consistently achieves competitive performance across multiple scenes and floors, outperforming representative models such as PointNet, PointNet++, DGCNN, and PCT under the same training and inference protocol. This indicates that the proposed architectural modules provide robust and transferable feature representations that generalize well across different structural layouts and scanning conditions, rather than being tailored to a specific scene or acquisition setup.

From the perspective of model architecture, the Fusion model combines the local geometric encoding of PointNet++ with the global self-attention modeling of PCT. The overall metric heatmap shows that this structure typically achieves high normalized scores across different scanning densities and viewpoint combinations. Component-level IoU statistics also reflect that the Fusion model maintains balanced recognition performance in most scenes, particularly for key components such as walls/columns, floors, and ceilings. It is evident that the local branch plays a role in fine-scale areas, such as beam-column connections and wall-panel junctions, while the global branch helps maintain semantic consistency under conditions of complex occlusion and viewpoint changes. The synergy between these two branches positively impacts the model’s stable performance under cross-domain conditions.

This observation is consistent with existing literature showing that hybrid architectures integrating local neighborhood features with global contextual modeling tend to outperform single-branch networks under varying point density, occlusion, and viewpoint conditions. Compared with purely local models, which are more sensitive to sparsity, and purely global attention-based models, which may overlook fine geometric details, the Fusion model exhibits a more balanced behavior in complex construction environments.

The inter-class feature correlation map provides another perspective on the analysis from the feature space. Overall, Fusion model exhibits lower cross-category correlation between multiple component groups, suggesting that the model can maintain some degree of category separation in the feature space. However, in some scenes, a higher local correlation is observed between Wall/column and “Other” categories, resulting in a small amount of mutual misclassification. This may be due to the simplified component types in virtual scanning scenes: BIM models mainly consist of structural components, while non-structural objects like curtains, furniture, and temporary materials in real scenes are typically classified as “Other” and are often placed adjacent to walls, causing their geometric distribution to be similar. This leads to blurred feature boundaries between these two categories in the real domain. The virtual domain lacks these components, which prevented the model from adequately learning this semantic difference during training.

The “Other” class in this study is defined as a broad residual category covering non-structural and temporary objects not explicitly represented in the BIM models, such as furniture, temporary materials, and site installations. This coarse definition introduces high intra-class variability and ambiguous geometric characteristics, which complicate the interpretation of misclassification patterns, particularly near dominant structural components like walls and columns. Similar confusion patterns between structural components and heterogeneous “Other” categories have been widely reported in real-world indoor and construction point cloud benchmarks, indicating that this issue reflects a common challenge in component-level semantic segmentation rather than a limitation specific to the proposed framework.

From a scalability perspective, the current framework demonstrates favorable behavior in handling large-scale point clouds through sliding-window partitioning and block-wise inference, which enables practical deployment on scenes containing billions of points. However, as scene scale and structural heterogeneity further increase, such as in industrial facilities or large public infrastructures, the computational cost associated with dense window sampling and multi-branch feature extraction may grow substantially. Moreover, highly heterogeneous scenes that involve diverse component types, complex functional layouts, and frequent temporary installations may introduce semantic patterns that are insufficiently represented in the current BIM-derived synthetic training set.

The above analysis highlights several directions for further improvement in this study. First, the synthetic point cloud generation process remains partially idealized, particularly with respect to material properties, small ancillary components, and temporary site facilities, which may introduce domain discrepancies in the “Other” category and certain elongated components. To address this issue, future work will focus on refining the semantic class taxonomy by decomposing both structural and non-structural elements into more fine-grained categories. For example, MEP-related components such as pipelines, as well as common furniture items (e.g., beds, tables, and cabinets), will be explicitly modeled rather than being uniformly absorbed into the “Other” class. Supported by richer BIM representations or auxiliary annotations, such semantic refinement is expected to reduce intra-class ambiguity, improve component-level discrimination, and enhance the diagnostic value of segmentation results in complex indoor construction environments.

Second, the empirical evaluation in this study is primarily based on self-acquired scans from a specific high-rise residential building, complemented by multiple scenarios from the BIMNet benchmark. Although multi-view scanning, sliding-window partitioning, and the inclusion of public datasets were employed to increase data diversity, the coverage of building typologies, structural systems, and construction stages remains limited. Consequently, while robust performance has been demonstrated within the evaluated scenarios, further validation is required to assess the generalization of the proposed framework to other building types, such as industrial or commercial facilities, which is left for future investigation.

## 6. Conclusions

This study demonstrates that BIM-generated synthetic point clouds can effectively support component-level semantic segmentation in real construction environments. Without using any real annotations, the Fusion model trained solely on SPC achieves 70.89% overall accuracy, 53.14% mean IoU, 69.67% mean accuracy, 54.75% FWIoU, and 59.66% Cohen’s κ, confirming the feasibility of annotation-free synthetic supervision for real-world scene understanding within the evaluated residential building scenarios and the BIM-Net benchmark.

The large-scale SPC dataset (132 scans, ~$8.75\times 10^{9}$ points) provides extensive geometric diversity and scanning variability. Across both scene-level and component-level evaluations, the Fusion model consistently exhibits leading or near-leading performance, reflecting stable semantic discrimination under varying floors, viewpoints, and scanning conditions in the tested residential buildings. This robustness highlights the effectiveness of the proposed global–local fusion strategy in mitigating domain discrepancies between synthetic and real point clouds.

Overall, the proposed framework exhibits strong potential for practical deployment in large-scale point cloud–based construction applications. Its extension to highly heterogeneous or industrial-scale environments, however, will require careful consideration of computational efficiency and enhanced semantic coverage beyond the current BIM-derived synthetic training set.

While the results underscore robust performance within residential settings, the framework’s generalization to more heterogeneous typologies—such as industrial or commercial complexes—is presently constrained by the semantic diversity and completeness of the underlying BIM representations. This limitation warrants further investigation to establish the framework’s broader applicability across the AEC (Architecture, Engineering, and Construction) industry. Addressing this limitation will require not only larger synthetic datasets but also richer semantic modeling of functional components and construction-specific objects that vary significantly across different building types.

Based on the current analysis, future work can be further advanced in the following directions: (i) constructing a richer BIM–Reality joint dataset by incorporating non-structural components such as windows, curtains, furniture, and MEP pipelines, to better narrow the semantic gap between the virtual and real domains; (ii) further refining the semantic class taxonomy by decomposing the coarse “Other” category into more fine-grained structural and non-structural component classes (e.g., MEP pipelines and common furniture), so as to reduce intra-class ambiguity and improve component-level recognition; (iii) introducing adversarial, contrastive, or self-supervised cross-domain alignment strategies at the model level to enhance adaptation to feature distribution shifts; (iv) developing hierarchical or coarse-to-fine inference strategies to reduce computational overhead and improve scalability in extremely large scenes; (v) exploring multi-modal fusion (e.g., images, depth maps, textures) to improve the distinguishability of geometrically similar components; (vi) embedding the segmentation results from virtual to real into the BIM dynamic updating process for automatic change detection, component status representation, and progress tracking, contributing to the formation of a closed-loop system for construction monitoring.

## Figures and Tables

**Table 1:** Summary of dataset composition and usage in this study.

Item	Synthetic Dataset (SPC)	Real-World Dataset
Data source	BIM-generated point clouds	Original LiDAR scan point clouds
Data scale	∼6×382 rooms, ∼6×8710 m^2^ floor area	382 rooms, ∼8710 m^2^ floor area
Number of scenes	132 synthetic scenes	16 representative real scenes
Generation/acquisition	Virtual LiDAR scanning using HELIOS++ [14]	Real on-site LiDAR scanning
Annotation	Automatically generated from BIM semantics	Provided by the original dataset
Usage in this study	Training only	Validation/Testing only

**Table 2:** Virtual Scanning Parameter Combinations (Synthetic LiDAR Scanning Network from BIM Model).

Experiment ID	Pulse Frequency (Hz)	Scanning Frequency (Hz)
exp1	50,000	240
exp2	50,000	360
exp3	100,000	240
exp4	100,000	360
exp5	500,000	240
exp6	500,000	360

## Data Availability

The data that support the findings will be available in [Dataset and benchmark for as-built BIM reconstruction from real-world point cloud] at [https://doi.org/10.1016/j.autcon.2025.106096] following an embargo from the date of publication to allow for commercialization of research findings.

## Abbreviations

The following abbreviations are used in this manuscript:

BIM	Building Information Modeling
V2R	Virtual-to-Real
SPC	Synthetic Point Cloud(s)
PCT	Point Cloud Transformer
OA	Overall Accuracy
mIoU	Mean Intersection-over-Union
FWIoU	Frequency-Weighted IoU
mAcc	Mean Accuracy

## Appendix A. Implementation Details

This appendix summarizes the low-level implementation details and training hyperparameters used in this study for reproducibility and completeness.

### Appendix A.1. Training Configuration

All models were trained using distributed multi-GPU parallelism with automatic mixed precision (AMP) enabled to improve computational efficiency while maintaining numerical stability. The AdamW optimizer was adopted for parameter optimization, combined with the OneCycleLR learning rate scheduling strategy. Exponential Moving Average (EMA) with a decay factor of α=0.99 was applied during training to stabilize parameter updates and enhance generalization performance.

The main training hyperparameters are listed as follows:

- Initial learning rate: $2\times 10^{-3}$
- Weight decay: $1\times 10^{-4}$
- Batch size: 8
- Gradient accumulation steps: 4
- Total training epochs: 100
- Warm-up epochs: 15
- Maximum number of points per sample: 8192
- Coordinate normalization: enabled

### Appendix A.2. Loss Function Settings

The training objective combines standard cross-entropy loss and Focal Loss to balance overall classification stability and hard-sample sensitivity. The Focal Loss focusing parameter was set to γ=2.0, and the final loss was computed as a weighted sum:

(A1) $$L=\left(1-\lambda \right)L_{\mathrm{ce}}+\lambda L_{\mathrm{focal}},\;\lambda =0.3.$$

### Appendix A.3. Data Augmentation and Regularization

In addition to conventional geometric augmentations (random rotation, scaling, translation, and jittering), an adaptive augmentation strategy based on PointAugment was employed. The augmentation network was constrained by a geometric consistency loss with weight $\lambda_{\mathrm{geom}}=0.1$ and a consistency loss with weight $\lambda_{\mathrm{cons}}=0.2$ to align synthetic and real feature distributions.

To further improve robustness to local structural variations, a probabilistic spatial occlusion mechanism was applied during training, with an occlusion ratio of 0.2, preventing the model from over-relying on dominant local regions.

## Appendix B. Quantitative Evaluation Results

**Table A1:** Performance across building datasets under different network backbones (unit: %). **Bold** and underline represent the best and second-best results in each row for each metric, respectively. * Data sourced from the BIMNet dataset.

Dataset	PointNet					PointNet++					DGCNN					PCT					Fusion				
	OA	mIoU	mAcc	FWIoU	κ	OA	mIoU	mAcc	FWIoU	κ	OA	mIoU	mAcc	FWIoU	κ	OA	mIoU	mAcc	FWIoU	κ	OA	mIoU	mAcc	FWIoU	κ
Ours	67.0	45.2	62.6	52.0	55.9	81.6	60.9	72.1	71.9	74.7	82.5	61.0	70.5	72.0	75.4	74.7	42.1	56.7	57.3	63.3	**83.7**	**64.0**	**73.9**	**75.3**	**77.6**
1px *	40.7	28.7	50.9	20.3	22.1	69.7	46.8	70.0	54.9	54.0	55.9	44.7	65.7	38.4	38.9	42.7	28.7	54.8	20.7	26.3	**71.9**	**50.9**	**74.4**	**57.3**	**57.5**
7y3 *	61.8	37.9	55.4	41.1	46.3	76.9	**57.2**	**71.1**	61.6	68.2	74.4	55.9	68.5	58.2	64.1	63.3	37.6	56.5	41.6	48.5	**77.2**	55.0	69.1	**62.2**	**68.8**
759 *	31.5	22.7	48.4	13.8	17.0	70.0	51.1	71.2	56.0	54.9	60.3	50.2	70.8	46.1	45.7	32.0	22.7	52.3	13.5	19.9	**71.6**	**55.5**	**75.6**	**57.7**	**58.2**
ac2 *	41.7	37.5	66.6	21.0	22.9	70.8	60.6	78.0	56.6	55.8	57.5	57.8	76.0	40.5	40.3	41.1	32.8	68.6	18.7	24.4	**73.1**	**63.9**	**81.8**	**58.6**	**59.4**
e9z *	41.3	32.9	52.4	23.9	22.3	69.6	60.4	74.0	56.8	58.2	63.1	56.3	70.9	47.9	49.1	45.7	33.8	61.0	24.3	29.5	**73.9**	**63.8**	**77.7**	**60.3**	**63.1**
hxp *	28.5	29.5	68.7	11.0	17.3	**66.9**	**50.0**	66.7	**54.3**	**40.8**	50.4	47.9	**69.2**	38.3	28.9	26.7	27.5	59.3	10.5	14.2	63.0	49.2	67.8	50.5	37.3
i5n *	35.5	35.4	60.6	19.0	18.7	65.9	55.6	76.7	51.2	51.6	53.7	51.1	74.9	35.0	38.0	39.7	32.3	67.5	18.2	24.7	**68.9**	**59.6**	**78.6**	**54.0**	**54.3**
i5n_1 *	41.8	37.8	69.2	19.9	22.7	61.6	51.5	72.7	45.1	42.4	55.4	51.4	72.8	37.4	36.2	41.5	34.7	64.5	19.4	21.8	**63.1**	**54.0**	**73.4**	**47.0**	**43.6**
px4_1 *	47.1	37.3	61.9	26.1	28.3	70.8	61.0	77.0	56.1	58.5	62.3	57.6	74.0	45.3	47.2	48.1	37.8	66.0	25.9	31.0	**74.5**	**65.4**	**80.8**	**60.3**	**63.6**
s9h *	42.9	30.8	51.4	23.9	27.9	69.9	50.3	76.9	56.1	56.0	61.4	50.8	69.6	47.2	47.3	42.5	31.8	52.8	23.4	28.4	**70.4**	**53.4**	**77.0**	**56.6**	**57.1**
sn8 *	48.1	41.3	65.4	27.6	31.5	69.1	58.5	72.6	54.4	55.2	63.9	59.7	74.0	48.8	49.4	49.4	41.5	67.3	28.0	33.8	**72.0**	**63.2**	**76.2**	**57.9**	**59.2**
skl *	51.9	40.8	64.9	30.3	31.8	67.2	56.4	72.7	51.9	52.8	62.2	54.6	69.9	45.9	45.1	52.1	38.5	64.2	29.8	32.2	**68.6**	**58.0**	**74.3**	**52.9**	**54.4**
st4 *	48.7	38.3	60.9	28.0	27.7	63.7	54.3	72.0	48.2	48.5	61.8	56.4	73.7	44.9	45.6	50.5	38.0	68.0	27.7	32.8	**67.9**	**58.7**	**76.4**	**52.1**	**53.8**
ur6 *	63.8	48.6	65.4	45.4	49.4	67.5	55.5	68.5	54.5	56.5	68.4	58.7	69.7	56.3	57.6	63.5	45.9	65.5	43.2	49.1	**73.1**	**62.8**	**74.9**	**60.2**	**63.4**
vvo *	49.1	32.4	56.4	30.4	32.8	71.2	50.3	74.1	56.1	61.3	64.8	46.7	64.8	48.7	52.9	53.4	32.1	51.6	31.8	38.9	**75.9**	**57.8**	**78.5**	**62.2**	**67.6**

**Table A2:** Per-component IoU comparison across building datasets (unit: %). W: Wall&Column, B: Beam, C: Ceiling, F: Floor, O: Other. Datasets marked with * are from the BIM-Net benchmark. **Bold** and underline represent the best and second-best results in each row for each metric, respectively. * Data sourced from the BIMNet dataset.

Dataset	PointNet					PointNet++					DGCNN					PCT					Fusion				
	W	B	C	F	O	W	B	C	F	O	W	B	C	F	O	W	B	C	F	O	W	B	C	F	O
Ours	54.4	24.8	64.2	62.2	**20.4**	77.1	57.4	86.3	73.0	10.5	75.4	47.1	90.4	**80.5**	11.4	69.0	0.4	78.0	62.9	0.1	**78.6**	**63.9**	**92.8**	73.8	10.6
1px *	31.3	0.0	57.9	53.8	0.3	43.9	1.5	71.6	60.8	56.4	37.6	3.5	**83.1**	**76.4**	22.9	33.3	0.1	59.5	50.0	0.3	**45.1**	**3.6**	79.6	68.9	**57.1**
7y3 *	52.7	0.7	58.5	76.4	1.0	**70.0**	**25.1**	68.2	80.0	42.8	64.4	21.9	**75.9**	**88.6**	28.6	56.1	0.4	58.5	72.6	0.3	69.9	5.2	66.1	84.5	**49.2**
759 *	21.5	4.3	35.2	51.0	1.4	35.0	38.7	48.9	70.9	**62.1**	28.0	38.1	**62.3**	**79.5**	43.2	21.3	0.7	35.7	55.4	0.3	**37.3**	**44.6**	58.4	75.5	61.6
ac2 *	33.0	0.0	57.1	57.6	2.3	50.7	0.0	71.7	64.2	55.9	40.3	0.0	**81.4**	**82.9**	26.7	34.0	0.0	49.3	47.7	0.3	**52.6**	**0.0**	76.4	70.0	**56.8**
e9z *	30.9	0.0	54.7	42.2	3.8	53.3	0.0	69.7	66.4	52.1	44.3	0.0	**78.5**	67.6	34.6	35.0	0.0	49.9	49.9	0.3	**53.4**	**0.0**	74.0	**70.2**	**57.5**
hxp *	17.8	0.0	48.2	51.8	0.2	**23.8**	0.0	60.8	54.6	**60.7**	20.3	0.0	**67.7**	**67.5**	35.9	18.7	0.0	54.6	36.4	0.3	21.8	**0.0**	62.9	57.0	55.0
i5n *	23.9	0.0	58.6	58.0	1.1	**45.3**	0.0	**75.9**	52.3	48.7	36.3	0.0	70.9	**78.3**	18.7	28.9	0.0	53.3	46.6	0.2	42.2	**0.0**	73.9	69.3	**53.0**
i5n_1 *	33.3	0.0	62.9	54.9	0.1	**40.1**	0.0	66.6	56.8	42.7	39.4	0.0	**74.2**	**66.0**	26.0	35.5	0.0	54.9	48.1	0.3	39.0	**0.0**	71.2	60.1	**45.5**
px4_1 *	38.5	0.0	53.7	55.8	1.2	54.6	0.0	72.7	67.0	49.9	45.6	0.0	**80.1**	**77.7**	27.0	38.1	0.0	57.3	55.5	0.3	**57.5**	**0.0**	77.5	72.0	**54.4**
s9h *	28.6	1.3	59.0	62.5	2.6	38.0	15.7	69.7	71.0	**57.3**	34.0	**23.0**	**80.1**	**80.7**	36.2	28.0	3.1	64.4	63.5	0.3	**38.0**	20.4	77.5	76.0	55.1
sn8 *	36.1	0.0	65.7	62.9	0.6	42.1	0.0	76.0	62.0	53.8	40.6	0.0	**83.7**	**79.0**	35.3	37.3	0.0	71.4	56.8	0.4	**43.9**	**0.0**	81.2	71.6	**56.2**
skl *	42.4	0.0	53.2	67.4	0.2	**48.9**	0.0	59.6	70.4	46.8	46.5	0.0	**65.0**	**76.9**	30.2	44.1	0.0	48.9	60.5	0.4	48.3	**0.0**	62.0	73.2	**48.5**
st4 *	39.8	0.0	49.3	62.3	2.0	44.4	0.0	66.3	64.5	41.9	45.0	0.0	**76.5**	**77.0**	27.2	41.7	0.0	55.2	54.8	0.4	**46.6**	**0.0**	71.6	69.9	**46.6**
ur6 *	51.6	0.0	65.5	75.8	1.6	58.7	0.0	55.2	71.8	36.3	57.1	0.0	62.8	**80.7**	34.2	50.7	0.0	65.3	67.2	0.3	**59.9**	**0.0**	**73.4**	79.9	**37.8**
vvo *	38.9	13.3	39.7	68.3	2.1	56.7	19.8	56.0	67.4	51.8	47.4	12.1	70.0	76.2	28.1	39.7	1.4	64.2	54.9	0.3	**58.7**	**23.3**	**74.7**	**79.8**	**52.6**
